# NLRP3-Inflammasome Inhibition during Respiratory Virus Infection Abrogates Lung Immunopathology and Long-Term Airway Disease Development

**DOI:** 10.3390/v13040692

**Published:** 2021-04-16

**Authors:** Carrie-Anne Malinczak, Charles F. Schuler, Angela J. Duran, Andrew J. Rasky, Mohamed M. Mire, Gabriel Núñez, Nicholas W. Lukacs, Wendy Fonseca

**Affiliations:** 1 Department of Pathology, University of Michigan, Ann Arbor, MI 48109, USA; carrieam@umich.edu (C.-A.M.); angduran@umich.edu (A.J.D.); arasky@med.umich.edu (A.J.R.); mmire@umich.edu (M.M.M.); bclx@med.umich.edu (G.N.); nlukacs@med.umich.edu (N.W.L.); 2 Department of Internal Medicine, Division of Allergy and Clinical Immunology, University of Michigan, Ann Arbor, MI 48109, USA; schulerc@med.umich.edu; 3 Mary H. Weiser Food Allergy Center, University of Michigan, Ann Arbor, MI 48109, USA

**Keywords:** NLRP3-inflammasome, early-life RSV, asthma, MCC950, IL-1β, respiratory virus, lung innate viral immunity

## Abstract

Respiratory syncytial virus (RSV) infects most infants by two years of age. It can cause severe disease leading to an increased risk of developing asthma later in life. Previously, our group has shown that RSV infection in mice and infants promotes IL-1β production. Here, we characterized the role of NLRP3-Inflammasome activation during RSV infection in adult mice and neonates. We observed that the inhibition of NLRP3 activation using the small molecule inhibitor, MCC950, or in genetically modified NLRP3 knockout (*Nlrp3−/−*) mice during in vivo RSV infection led to decreased lung immunopathology along with a reduced expression of the mucus-associated genes and reduced production of innate cytokines (IL-1β, IL-33 and CCL2) linked to severe RSV disease while leading to significant increases in IFN-β. NLRP3-inflammasome inhibition or deletion diminished Th2 cytokines and inflammatory cell infiltration into the lungs. Furthermore, NLRP3 inhibition or deletion during early-life RSV infection led to reducing viral-exacerbated allergic response in a mouse model of RSV-induced allergy exacerbation. Here, we demonstrated the critical role of NLRP3-inflammasome activation in RSV immunopathology and the related long-term airway alteration. Moreover, these findings suggest the NLRP3-inflammasome as a potential therapeutic target to attenuate severe RSV disease and limit childhood asthma development.

## 1. Introduction

RSV infection is the leading cause of childhood hospitalization and increases the risk for developing childhood asthma and recurrent wheezing [1,2]. RSV can skew the immune response away from antiviral and towards a Th2-type response by inhibiting the production of type 1 interferon (IFN) [3]. Severe RSV disease has been linked with a strong Th2 immune response that led to lung pathologies, such as allergy and asthma, by promoting an inflammatory cellular environment [4,5,6].

An important source of Th2 cytokines are the innate lymphoid 2 cells (ILC2). Increased numbers of these cells have been observed during RSV infection and have been related with the development of lung inflammation and mucus production [7,8]. The ILC2 are activated directly by epithelium-associated cytokines IL-25, IL-33 and TSLP [9], which are strong drivers of Th2 immune responses [10]. It has also been shown that IL-1β is a critical activator of ILC2 since it can regulate the expression of epithelial cytokine receptors, promote proliferation and cytokine production [11]. Previously, our group has shown that RSV infection in mice and infants promotes IL-1β pathway activation, which leads to the induction of innate cytokines (CCL2, TSLP and IL-33) by the airway epithelium [12] and that the inhibition of the IL-1β pathway with IL-1 receptor antagonist during early-life RSV infection altered RSV immunopathology and diminished subsequent exacerbated allergic disease [13].

NLRP3-inflammasome is a multiprotein complex, which consists of NLRP3 (nucleotide-binding domain leucine-rich repeat (NLR) and pyrin domain-containing receptor 3), the adaptor ASC (apoptosis-associated speck-like protein containing a caspase recruitment domain) and procaspase-1 [14,15,16]. NLRP3-inflammasome plays a crucial role in innate immunity that elicits the release of proinflammatory cytokines such as IL-1β and IL-18 [15,16]. In mice, activation of NLRP3 inflammasome in macrophages requires two steps: (1) priming (signal 1) is provided by inflammatory stimuli such as TLR4 agonists which induce NF-κB-mediated NLRP3 and pro-IL-1β expression and activation and (2) the activation step (signal 2) that is triggered by certain pathogen-associated molecular patterns (PAMPs) such as viral RNA, particulate matter, toxins and damage-associated molecular patterns (DAMPs) that promote NLRP3 inflammasome assembly and caspase-1-mediated IL-1β and IL-18 secretion and pyroptosis [15,17]. RSV infection upregulated NLRP3-inflammasome [18,19] and activated caspase-1 [20], both events are crucial for IL-1β production during RSV infection [18]. Finally, RSV small hydrophobic protein (RSV SH) can activate the NLRP3- inflammasome [19]. The studies presented here used a direct NLRP3 inhibitor, MCC950, a potent and selective NLRP3 inflammasome inhibitor that blocks caspase 1 processing of IL-1, that is being considered for clinical trials [16,21].

The current study characterized the direct role of NLRP3-inflammasome in the development of RSV immunopathology and the long-term alteration in the lung immune environment. Here, we demonstrate that by inhibiting NLRP3-Inflammasome activity by using a specific inhibitor (MCC950) or NLRP3 knockout mice, the RSV immunopathology is abolished as well as subsequent allergic exacerbation following early-life RSV. These studies further suggest the NLRP3-inflammasome as a possible therapeutic target to mitigate RSV immunopathology and its associated long-term airway alterations. 

## 2. Materials and Methods

### 2.1. Mice Models

All experiments involving the use of animals were approved by the University of Michigan Care and Use of Animals Committee. Female and male (BALB/c, C57BL/6) mice of 6 to 8 weeks of age were purchased from The Jackson Laboratory (Bar Harbor, ME, USA). *Nlrp3−/−* mice on C57BL/6 background have been reported [22]. For adult infection mice model or for in house breeding. All mice were maintained under standard pathogen-free conditions. 

#### 2.1.1. Adult Mice Model

Female BALB/c mice 6–7 weeks old were intratracheally infected with 50 μL/mouse of RSV (2 × 10^5^ pfu) [23] Line 19 antigenic subgroup A. The mice were treated intraperitoneally with MCC950 (Cayman Chemicals, Ann Arbor, MI, USA) (20 mg/kg/mouse), or with vehicle (EtOH final concentration 0.5%) every day during the time of infection. On Day 6 post-infection, we collected samples for flow cytometry and bronchoalveolar lavages (BAL) and on Day 8 post-infection, we collected samples for histology and RNA extraction.

#### 2.1.2. Neonatal Model

Female and male neonatal mice from in house breeding colony, BALB/c, C57BL/6, or *Nlrp3−/−* mice and intranasally infected with 5 μL/mouse of RSV (1 × 10^5^ pfu) Line 19 antigenic subgroup A at 7 days of age as described previously [24]. The mice were treated intraperitoneally with MCC950 (20 mg/kg/mouse) or with vehicle (EtOH final concentration 0.5%) every day during the time of infection. On Day 6 post-infection, we collected samples for flow cytometry and on Day 8 post-infection, we collected samples for histology and RNA extraction. 

#### 2.1.3. RSV/CRA Model

Neonatal mouse model was performed, followed by CRA allergy model.

Cockroach allergen (CRA) (HollisterStier Allergy, Spokane, WA, USA) was administered via intratracheal instillation into the lungs over 3 consecutive days, starting at 4-weeks post-infection, followed by 4 challenges 2 weeks later to elicit an allergic response as previously described [24]. As a control, we have naïve and CRA only treated groups. In brief, mice were sensitized intratracheally with 500 protein nitrogen units (pnu) of CRA, over 3 consecutive days. Next, mice were challenged at Days 14, 20, 22 and 23 with 500 pnu of CRA. Samples were harvested on Day 24. 

### 2.2. Respiratory Syncytial Virus

Our laboratory uses antigenic subgroup A, Line 19 RSV, obtained initially from a sick infant at the University of Michigan Hospital System. This isolate has been shown to mimic human infection by eliciting airway mucus production in animal models [23]. 

### 2.3. Quantitative RT-PCR

Lung tissue was homogenized in TRIzol reagent (Invitrogen, Carlsbad, CA, USA) to extract RNA cDNA was synthesized using murine leukemia virus reverse transcriptase (Applied Biosystems, Foster City, CA, USA) and incubated at 37 °C for one hour followed by incubation at 95 °C for 5 min to stop the reaction. Real-time quantitative PCR (qPCR) was multiplexed using Taqman primers, with a FAM-conjugated probe and GAPDH with a VIC-conjugated probe (Applied Biosystems, Foster City, CA, USA) to measure *Ifnb, Il4, Il5* and *Il13* gene expression. Fold change was quantified using the 2^−**ΔΔ**^ cycle threshold (CT) method. Custom primers were designed to measure RSV F gene, *Muc5ac* and *Gob5* mRNA levels as described [13]. All reactions were run on a 7500 Real-Time PCR System (Applied Biosystems, Foster City, CA, USA).

### 2.4. Lung Histology

The left lung was perfused with 4% (*v/v*) formaldehyde for fixation and embedded in paraffin. Five-micrometer lung sections were stained with periodic acid-Schiff (P.A.S., Middleton, WI, USA) to detect mucus production and inflammatory infiltrates were observed with hematoxylin and eosin stain (H&E, Middleton, WI, USA). Photomicrographs were captured using a Zeiss Axio Imager Z1 and Axio Vision 4.8 software (Zeiss, Munich, Germany).

### 2.5. Enzyme-Linked Immunosorbent Assays

Murine IL-1β and IL-33 proteins were quantified from Bronchial alveolar lavage (BAL) samples taken from naïve or infected mice after euthanasia. For IL-1β and IL-33, R&D Duo set ELISA kit was used (R&D Systems, Minneapolis, MN, USA) per the manufacturer’s instructions.

### 2.6. Protein Lung Extraction and Cytokine Quantification

Lung protein was extracted using a tissue cell lysis reagent T-PER (Thermo Fisher, Rockford, IL, USA), following the manufacturer’s instructions. The total protein was quantified using Pierce BCA assay kit (Thermo Fisher, Rockford, IL, USA) and the protein levels of IL-4, IL-5, IL-13, IL-17 and CCL2/MCP-1 were measured with a Bio-Plex cytokine assay (Bio-Rad Laboratories, Hercules, California, USA). 

### 2.7. Flow Cytometry 

The lungs were removed to analyzed leukocytes population. Lung single cells were isolated by enzymatic digestion with 1 mg/mL collagenase A (Roche, Indianapolis, IN, USA) and 20 U/mL DNase I (Sigma, St. Louis, MO, USA) in RPMI 1640 containing 10% fetal calf serum (FCS). Tissues were further dispersed through an 18-gauge needle (10-mL syringe), RBCs were lysed and samples were filtered through 100 μm nylon mesh twice. Cells were resuspended in PBS and live cells were identified using LIVE/DEAD Fixable Yellow Dead Cell Stain kit (Thermo Fisher Scientific, Waltham, MA, USA), then washed and resuspended in PBS with 1% FCS and Fc receptors were blocked with purified anti-CD16/32 (clone 93; BioLegend, San Diego, CA, USA). Surface markers were identified using Abs (clones) against the following antigens, all from BioLegend: anti-Gr-1 (RB6- 8C5), B220 (RA3-6B2), CD3 (145-2C11), Ter119 (Ter-119), CD11b (M1/70), CD25 (PC61), CD45 (30-F11), CD127 (SB/199), ST2 (DIH9), c-Kit (2B8), CD90 (53-2.1), CD4 (RM4-5), CD3 (17A2), CD8 (53-5.8), CD69 (H1.2F3) CD11c (N418), MHCII (M5/114.15.2), CD103 (2E7). SiglecF (E50-2440) was purchased from B.D. Biosciences (San Jose, CA, USA). For innate lymphoid cell staining, lineage markers were anti-CD3, CD11b, B220, Gr-1 and TER119. ILC2: Lin-CD45+ CD90+ST2+ c-Kit +CD127+GATA3+. Eosinophils: SSC^high^ CD11b+ SiglecF+. Neutrophils: SSC^high^ CD11b+ SiglecF- GR-1+. Antigen-presenting cells (APC): CD11b+CD11c+MHCII+, CD103-. CD103+APC: CD11c+MHCII+CD11b-CD103+. Interstitial macrophages: CD11b+CD11c-F4/80+. CD4+ T cells: CD3+CD4+, CD8+ T cells: CD3+CD8+. Data was collected using NovoCyte flow cytometer (ACEA Bioscience, Inc. San Diego, CA, USA). Data analysis was performed using FlowJo software (Tree Star, OR, USA). 

### 2.8. Statistical Analysis

Data were analyzed by Prism 8 (GraphPad Software, San Diego, CA, USA). Results are expressed as mean ± standard error. Statistical significance was measured by one-way or two-way ANOVA followed by post hoc Student’s *t*-test as appropriate. A *p* ≤ 0.05 was considered significant

## 3. Results

### 3.1. NLRP3-Inflammasome Inhibition Abrogates RSV Immunopathology in Adult Mice

**Figure 1:**
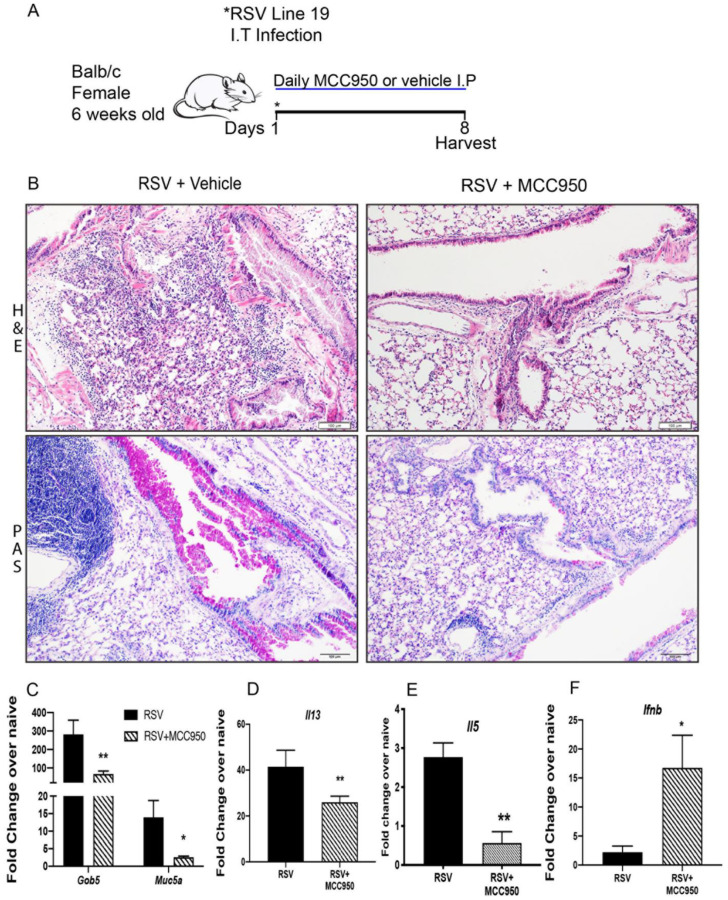
NLRP3-inflammasome inhibition abrogated RSV immunopathology in adult mice. (**A**) 6–7-week-old adult female BALB/c mice were infected intratracheally with Respiratory Syncytial Virus (RSV), Line 19 and treated daily with either an NLRP3 inhibitor (MCC950) or vehicle intraperitoneally; samples were harvested at 8 days post-infection. (**B**) Lung histopathology in Hematoxylin and Eosin stain (H&E) showed strongly reduced lung inflammatory infiltration and Periodic acid-Schiff stain (PAS) detected mucus that was decreased in the RSV+MCC950 group compared with control RSV+Vehicle. (**C**) Decreased *Gob5* and *Muc5ac* lung mRNA expression. (**D**) *Il13* and (**E**) *Il5* mRNA expression. (**F**) Upregulation of *Ifnb lung mRNA expression*. Data represent the Mean ± SEM from 4 to 5 mice (experimental repeats 2). * *p* ≤ 0.05, ** *p* ≤ 0.01.

It has been described that RSV infection upregulates IL-1β through the activation of NLRP3-inflammasome [12,13,18]. To characterize the role of NLRP3-inflammasome inhibition, we infected adult BALB/c mice with RSV Line 19 and treated them daily with either an NLRP3 inhibitor (MCC950) or vehicle (0.5% EtOH) (Figure 1A). The histopathological findings reveal that inhibition of NLRP3-inflammasome during RSV infection using MCC950, a direct inhibitor of the NLRP3 protein [16], strongly reduced lung inflammatory infiltration and decreased mucus deposition in the airways, when compared with infected controls (Figure 1B). MCC950 treatment correspondingly decreased mucus-related gene expression *Gob5* and *Muc5ac* (Figure 1C). Significantly decreased expression of mucus inducing *Il13* (Figure 1D) and the Th2 cytokine *Il5* (Figure 1E) with significant upregulation of antiviral *Ifnb* (Figure 1F) were also observed. No significant difference was observed in the expression of RSV fusion gene in the lung of the animals infected and treated when compared with infected controls (data not shown). These data suggest that NLRP3-inflammasome inhibition during RSV infection abrogates viral lung immunopathology and promotes innate antiviral responses by increasing type 1 interferon. 

### 3.2. RSV Immunopathology Is Dependent on NLRP3-Inflammasome Function 

**Figure 2:**
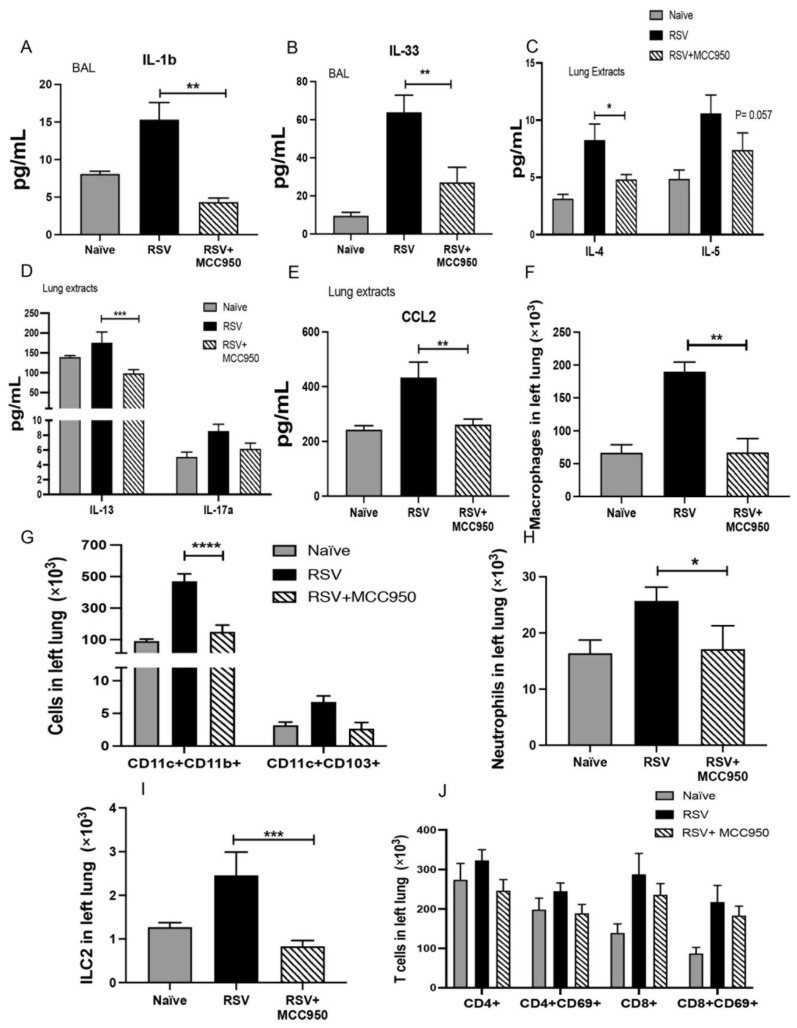
RSV immunopathology is dependent on NLRP3-inflammasome function Bronchoalveolar lavage (BAL) samples of adult mice were used to measure (**A**) IL-1β and (**B**) IL-33. Total lung extracts were used to evaluate levels of (**C**) IL-4 and IL-5, (**D**) IL-13 and IL-17 and (**E**) CCL2. (**F**–**J**) Flow cytometry of lung leukocytes. Data represent the Mean ± SEM from 4 to 5 mice (experimental repeats 2). * *p* ≤ 0.05, ** *p* ≤ 0.01, *** *p* ≤ 0.001, **** *p* ≤ 0.0001.

To characterize the role of NLRP3-inflammasome activation in the inflammatory immune response, we evaluated bronchoalveolar lavages (BAL) of adult mice infected with RSV under MCC950 treatment and control. First, we analyzed the production of IL-1β in the BAL of the adult mice and we observed that MCC950 treatment decreased levels of IL-1β in the sample when compared with control group (Figure 2A). Furthermore, NLRP3 inhibition diminished production of IL-33 in the BAL fluid compared with controls (Figure 2B). Levels of IL-4, IL-13 and CCL2, were significantly decreased in total lung protein extracts (Figure 2C,E), with no significant differences observed in IL-5 and IL-17 (Figure 2C,E). Total leukocyte numbers were analyzed to characterize the inflammatory response and we observed significantly reduced total numbers of interstitial macrophages, CD11c+CD11b+ antigen-presenting cells (APC), neutrophils and type 2 innate lymphoid cells (ILC2) in the MCC950 treated mice compared with control RSV infection (Figure 2F,I) with no difference in T cells (Figure 2J). These data suggest that NLRP3-inflammasome activity has an important role during RSV immune response, which leads to Th2 immune response and severe disease. 

### 3.3. NLRP3-Inflammasome Inhibition Modifies RSV Immunopathology in Neonate Mice 

**Figure 3:**
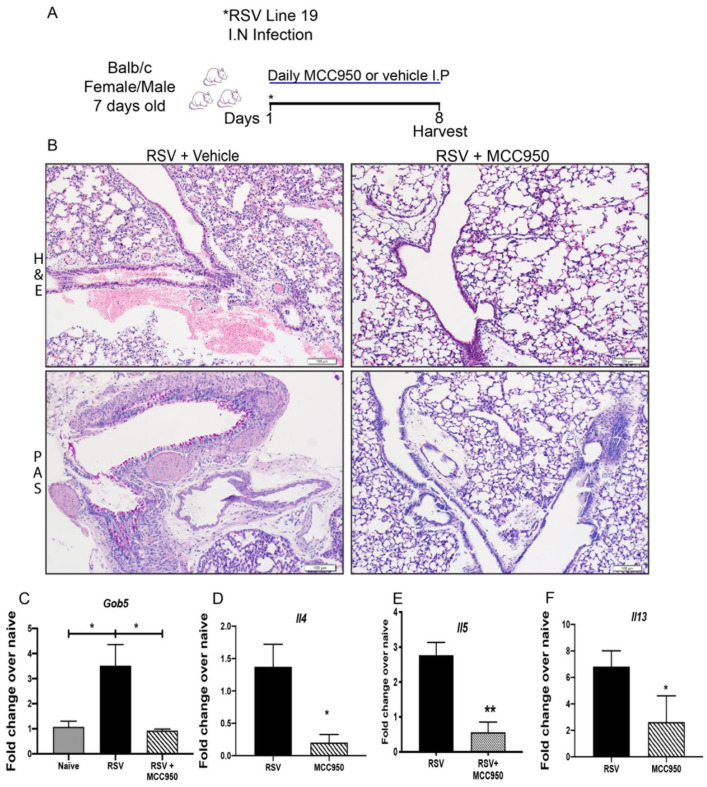
NLRP3-inflammasome inhibition modifies RSV immunopathology in neonate mice. (**A**) 7-day old female and male BALB/c mice were infected intranasally with Respiratory Syncytial Virus (RSV), Line 19 and treated daily with either an NLRP3 inhibitor (MCC950) or vehicle intraperitoneally; samples were harvested at 8 days post-infection. (**B**) Lung histopathology in Hematoxylin and Eosin stain (H&E) showed strongly reduced lung inflammatory infiltration and Periodic acid-Schiff stain (PAS) detected mucus that was decreased in the RSV+MCC950 group compared with control RSV+ Vehicle. Scale bar = 100 μm. (**C**) Decreased *Gob5* lung mRNA expression. (**D**) *Il4*, (**E**) *Il5*, (**F**) *Il13* lung mRNA expression. Data represent the Mean ± SEM from 4 to 8 mice (experimental repeats 2). * *p* ≤ 0.05, ** *p* ≤ 0.01.

**Figure 4:**
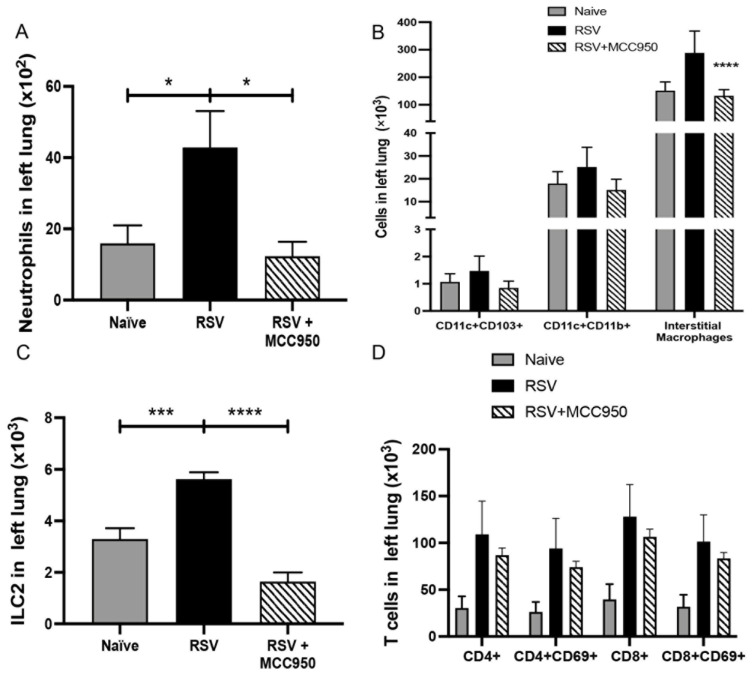
NLRP3-inflammasome inhibition modifies RSV inflammatory response in neonate mice. Neonatal mice were infected with RSV and treated with MCC950 or vehicle. Flow cytometry of the lung leukocytes: (**A**) Total neutrophils (**B**) Antigen-presenting cells (**C**) Type 2 innate lymphoid cells (ILC2) (**D**) T cells. Data represent the Mean ± SEM from 4 to 5 mice (experimental repeats 2). * *p* ≤ 0.05, *** *p* ≤ 0.001, **** *p* ≤ 0.0001.

The severity of RSV infection and immunopathology has been linked with different factors. For example, the age at which a child is infected, as preterm babies and infants are more susceptible to developing severe RSV disease [25]. To examine the role of NLRP3-inflammasome inhibition on RSV immunopathology, we intranasally infected neonate BALB/c mice at 7 days of age treated with MCC950 or vehicle (Figure 3A). Examination of histopathology determined that the MCC950 treatment reduced mucus production and goblet cell metaplasia compared with vehicle-treated controls (Figure 3B) as well as a correlative decrease in mucus gene expression of *Gob5* (Figure 3C). Importantly, a reduction of Th2 cytokine gene expression of *Il4*, *Il5* and *Il13* was observed in MCC950-treated mice (Figure 3D–F). Examination of lung inflammation demonstrated decreased numbers of neutrophils, interstitial macrophages, ILC2 and CD8+ T cells in the RSV+MCC950-treated group compared with controls (Figure 4A,D). Thus, similar to adults, neonatal mice treated with MCC950 during RSV infection presented decreased RSV immunopathology, suggesting the NLRP3-inflammasome plays an important role in the RSV severity during early-life.

### 3.4. Decreased RSV Immunopathology in Nlrp3−/− Neonate Mice

**Figure 5:**
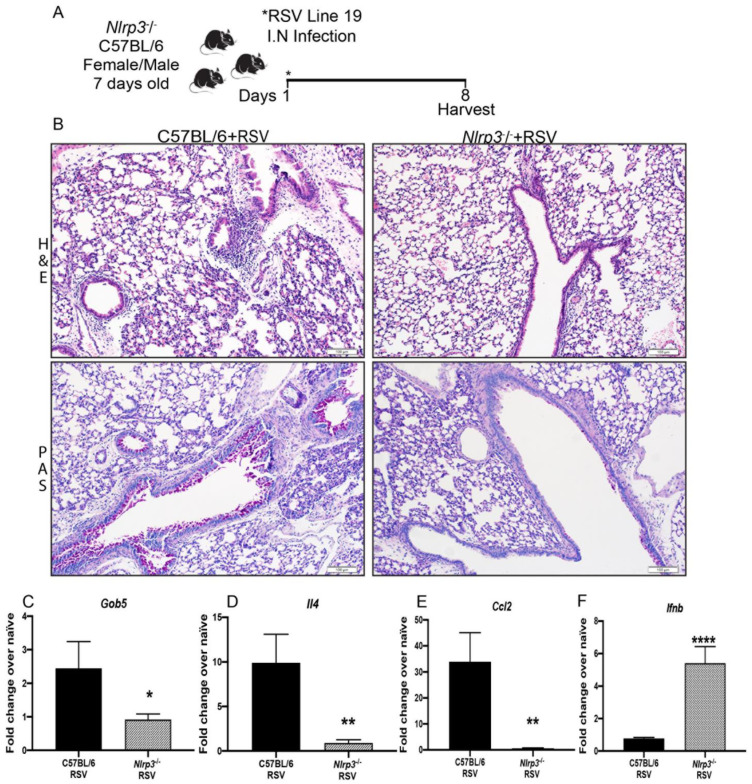
Decreased RSV immunopathology in *Nlrp3−/−* neonate mice. (**A**) 7-day old *Nlrp3−/−* or C57BL/6 neonatal mice were infected intranasally with Respiratory Syncytial Virus (RSV), Line 19. (**B**) Lung histopathology in Hematoxylin and Eosin stain (H&E) showed strongly reduced lung inflammatory infiltration and Periodic acid-Schiff stain (PAS) detected mucus that was decreased in the *Nlrp3−/−* mice compared with control C57BL/6. Scale bar = 100 μm. (**C**) Decreased *Gob5* lung mRNA expression and (**D**) *IL-4*, (**E**) *Ccl2*, (**F**) Upregulated lung expression of *Ifnb.* Data represent the Mean ± SEM from 4 to 8 mice (experimental repeats 2). * *p* ≤ 0.05, ** *p* ≤ 0.01, **** *p* ≤ 0.0001.

**Figure 6:**
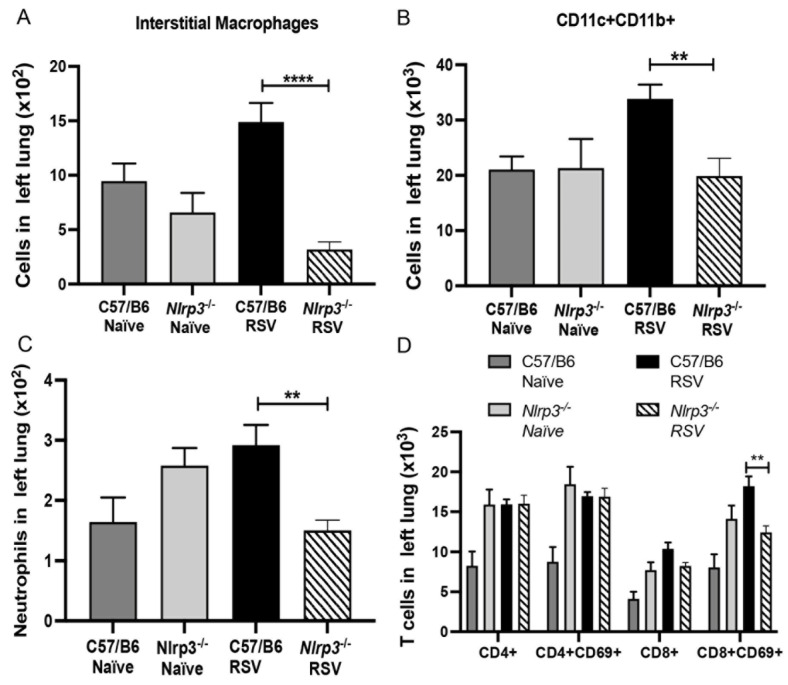
Diminished RSV airway inflammation in *Nlrp3−/−* neonate mice. (**A**) Neonatal *Nlrp3−/−* or C57BL/6 mice were infected with RSV. Flow cytometry of the lung leukocytes (**A**) Interstitial macrophages (**B**) CD11c+CD11b+ antigen-presenting cells (**C**) Neutrophils (**D**) T cells. Data represent the Mean ± SEM from 4 to 5 mice (experimental repeats 2). ** *p* ≤ 0.01, **** *p* ≤ 0.0001.

To further investigate the role of NLRP3 inflammasome during neonatal RSV infection, we used *Nlrp3−/−* neonatal mice and as a control, we used age-matched C57BL/6 mice (Figure 5A). The histopathological finding showed that *Nlrp3−/−* neonatal mice presented decreased inflammatory airway infiltration and reduced mucus deposition compared to C57BL/6 infected group (Figure 5B). Significant downregulation of the expression of *Gob5, Il4, Ccl2* and *Ifnb* was observed in *Nlrp3−/−* neonatal mice when compared with control mice (Figure 5C–F). Inflammatory lung infiltration was evaluated by flow cytometry and we observed that *Nlrp3−/−* mice presented significantly decreased numbers of interstitial macrophages, CD11c+CD11b+ APC, neutrophils and CD8+CD69+ T cells (Figure 6A,D). These data demonstrated the critical role of NLRP3-inflammasome in the RSV immunopathology in neonatal mice.

### 3.5. NLRP3-Inflammasome Inhibition during Early-Life RSV Infection Attenuated Secondary Allergic Exacerbation

**Figure 7:**
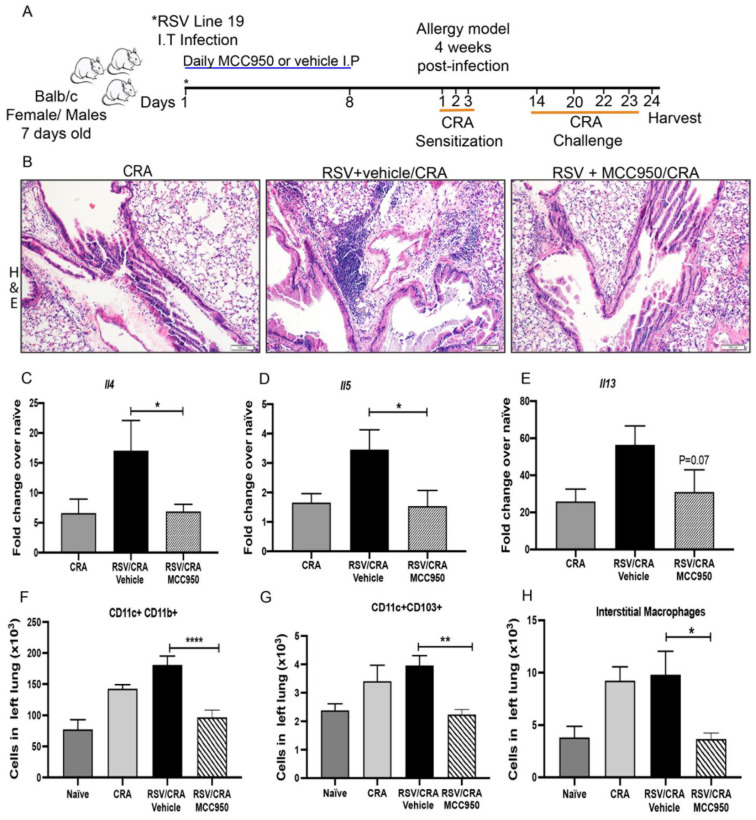
NLRP3-inflammasome inhibition during early-life RSV infection attenuated secondary allergic exacerbation. (**A**) 7-day old BALB/c neonate mice were infected with RSV, followed by cockroach allergy model (CRA). CRA was administered via intratracheal instillation into the lungs over 3 consecutive days, starting at 4-weeks post-infection, followed by 4 challenges 2 weeks later to elicit an allergic response as previously described. Naïve and CRA only treated mice were used as controls. (**B**) Lung histopathology in Hematoxylin and Eosin stain (H&E) showed strongly reduced lung inflammatory infiltration in the RSV+MCC950/CRA group compared with control RSV+ Vehicle/CRA. Scale bar = 100 μm. (**C**) *Il4*, (**D**) *Il5*, (**E**) *Il13* mRNA expression. Flow cytometry of the lung leukocytes (**F**) CD11c+CD11b+ antigen-presenting cells (**G**) CD11c+CD103+ antigen-presenting cells (**H**) Interstitial macrophages. Data represent the Mean ± SEM from 4 to 8 mice (experimental repeats 2). * *p* ≤ 0.05, ** *p* ≤ 0.01, **** *p* ≤ 0.0010.

Severe RSV infection is the most prominent cause of bronchiolitis and childhood hospitalization in infants under six months of age. This group of infants is the most susceptible to developing severe RSV disease and asthma later in life [1,2]. To evaluate whether the NLRP3-inflammasome during early-life impacts long term disease phenotypes, we used neonatal RSV infection followed by the cockroach allergic model (Figure 7A). Neonatal RSV infected mice treated with MCC950 or vehicle during the infection were sensitized and challenged with cockroach allergen (CRA) at 5 weeks of age. As controls, naïve and CRA only treated age-matched animals were used. Lung histology showed that inhibition of NLRP3 during early life RSV infection (RSV+MCC950/CRA) did not have an exacerbated allergic phenotype compared to the control treated neonatal RSV infected mice (RSV+vehicle/CRA) (Figure 7B). Lung cytokine gene expression of *Il4* and *ll-5* was significantly downregulated with a trend towards decreased *Il13* in the RSV+MCC950/CRA compared with RSV+vehicle/CRA group (Figure 7C,E). Inflammatory infiltration in the lung was evaluated and we observed significantly decreased numbers of APC, including CD11c+CD11b+, CD11c+CD103+ and interstitial macrophages in the RSV+MCC950/CRA when compared with RSV+vehicle/CRA (Figure 7E,G). Altogether, these data indicate that NLRP3-inflammasome has a role during early-life RSV infection that alters the immune response to the secondary lung insult in the adult mice. 

## 4. Discussion

The current studies demonstrate that inhibition of NLRP3-Inflammasome activity by a specific inhibitor (MCC950) or in NLRP3 knockout mice abrogated RSV immunopathology as well as the subsequent allergic airway exacerbation after early-life RSV infection. NLRP3-inflammasome is a multiprotein complex, which plays a crucial role in innate immunity that elicits the release of proinflammatory cytokines like IL-1β and IL-18 [15,16]. Increased levels of IL-1β has been linked with the severity of disease in various respiratory virus infections, including RSV [12,13,26], rhinovirus [27,28,29] and SARS-CoV2 [30,31,32]. Since aberrant activation of NLRP3-inflammasome and IL-1 are associated with numerous chronic diseases, including cardiovascular, atherosclerosis and diabetes, the interest in developing or identifying potent and specific NRLP3 inhibitors has been of increased interest [16]. Targeting NLRP3 activation could offer a safer choice to prevent immunosuppressive side effects that can arise by targeting other downstream products of the NLRP3-inflammasome, such as IL-1, IL-18, or IL-33 [16]. Importantly, targeting NLRP3-activation resulted in decreased IL-1β, IL-33 and CCL2 production with significantly increased type 1 IFN-β, suggesting that targeting NLRP3 rebalanced the antiviral immune response decreasing detrimental proinflammatory pathways. 

IL-1β and IL-33 belong to the IL-1 family of cytokines and receptors primarily associated with innate immunity [11,33,34]. It has been shown that IL-1β and IL-33 are critical activators of ILC2 that induce proliferation and cytokine production (e.g., IL-5 and IL-13) and also regulate the expression of epithelial cytokine receptors [11]. The inhibition of NLRP3-inflammasome during RSV infection in adult and neonate mice diminished the Th2 cytokine expression, likely related to the activation and proliferation of ILC2, which are a significant source of IL-13 in the lungs [9,35]. These data correlated with the decreased mucus production that has been associated with the production of IL-13 and the development of goblet cell hyperplasia [36]. IL-1β also impacts the airway epithelium immune homeostasis by increasing the production of IL-33 and CCL2 from airway epithelial cells [12,37]. Altogether, these data suggest that NLRP3-inflammasome inhibition impacts the Th2 immune response by modifying the activation and proliferation of ILC2 through the reduction of IL-1β and IL-33 in the airway. 

Previously, our group has shown that RSV infection in mice and infants promotes IL-1β pathway activation, which leads to the induction of innate cytokines (CCL2, TSLP and IL-33) by the airway epithelium [12,13]. Our previous studies have indicated that primary cultures of alveolar epithelial cells type II from naïve mice had increased expression of epithelial cell-derived cytokines IL-33, TSLP and CCL2 after in vitro infection with RSV [12]. We have also shown that early-life RSV infection generated persistent expression of IL-33 in alveolar epithelial cells and TSLP within the bronchial airway epithelial cells [24].

Here, we observed decreased production and expression of pulmonary CCL2 where NLRP3-inflammasome was inhibited (MCC950) or genetically abrogated in *Nlrp3−/−* mice. CCL2 is a potent chemoattractant of myeloid and lymphoid cells and it has been suggested that this chemokine can impact leukocyte phenotype, polarization, effector molecule secretion, autophagy and survival, in a context-dependent manner [38]. Abnormal upregulation of CCL2 results in sustained and exacerbated cell recruitment and uncontrolled inflammation [38], as seen in rheumatoid arthritis, atherosclerosis, diabetes and COVID-19, among others [39,40,41,42,43,44]. When NLRP3 was blocked during RSV infection a downregulation of CCL2 expression and diminished total numbers of antigen-presenting cells (CD11c+CD11b+ and interstitial macrophages) was observed. Thus, NLRP3 activation provides a central and early role for regulating the immunopathology response during RSV infection that may have long term effects.

**Figure 8:**
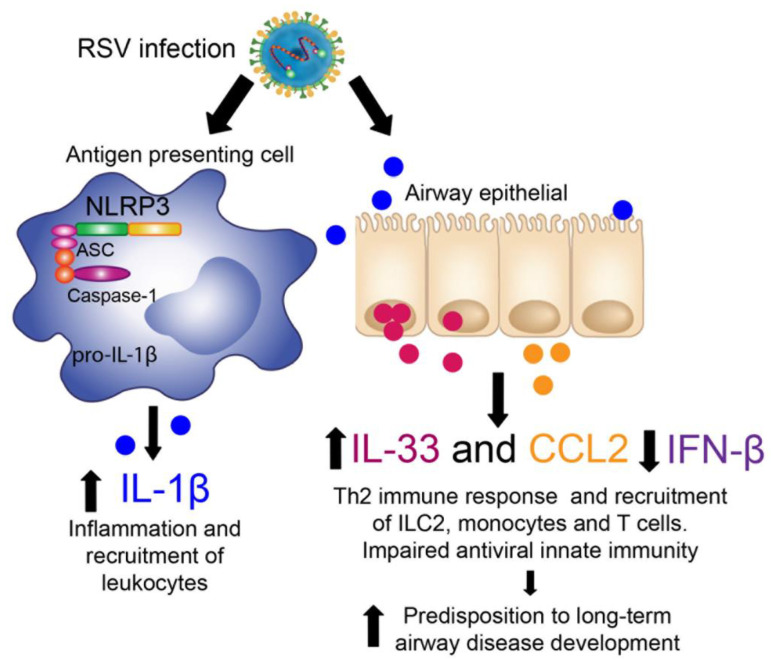
RSV induces NLRP3-inflammasome activation and leads to Th2 response. RSV infection activated NLRP3-inflammasome in Antigen presenting cells (APC), that trigger the production of IL-1β. Airway epithelial cells increases the expression of IL-33 and CCL2 when IL-1β is upregulated and downregulated IFNβ. IL-33 and CCL2 can recruit ILC2, monocytes, APC and T cells to the lung and increase the predisposition to airway disease development later in life.

Clinical studies have supported a link between early-life severe RSV infection and airway disease development later in life, including asthma [1,2,6]. In this work, we observed that inhibition of the NLRP3 inflammasome during early-life RSV infection impacts the subsequent response to a secondary insult (CRA) later in life by decreasing inflammatory lung infiltration, including APC that led to decreased lung Th2 cytokine phenotype (Figure 8). Similar results were observed using interleukin 1 receptor antagonist (IL-1ra) [13], corroborating the importance of this pathway. Multiple early life events in the lung have been associated with NLRP3 activation, including viral and bacterial infections, Bronchopulmonary dysplasia and even septic events, which have been associated with increased susceptibility to later disease [13,45,46,47]. Perhaps targeting NLRP3 during these early life events may provide a common therapeutic pathway for controlling long term sequelae that are often observed later in these children, including childhood asthma.

## References

[1] Sigurs N., Aljassim F., Kjellman B., Robinson P.D., Sigurbergsson F., Bjarnason R., Gustafsson P.M. (2010). Asthma and allergy patterns over 18 years after severe RSV bronchiolitis in the first year of life. Thorax.

[2] Henderson J., Hilliard T.N., Sherriff A., Stalker D., Al Shammari N., Thomas H.M. (2005). Hospitalization for RSV bronchiolitis before 12 months of age and subsequent asthma, atopy and wheeze: A longitudinal birth cohort study. Pediatr. Allergy Immunol..

[3] Swedan S., Andrews J., Majumdar T., Musiyenko A., Barik S. (2011). Multiple functional domains and complexes of the two nonstructural proteins of human respiratory syncytial virus contribute to interferon suppression and cellular location. J. Virol..

[4] Lotz M.T., Peebles R.S. (2012). Mechanisms of respiratory syncytial virus modulation of airway immune responses. Curr. Allergy Asthma Rep..

[5] Lambert L., Sagfors A.M., Openshaw P.J., Culley F.J. (2014). Immunity to RSV in Early-Life. Front. Immunol..

[6] Bacharier L.B., Cohen R., Schweiger T., Yin-Declue H., Christie C., Zheng J., Schechtman K.B., Strunk R.C., Castro M. (2012). Determinants of asthma after severe respiratory syncytial virus bronchiolitis. J. Allergy Clin. Immunol..

[7] Cormier S.A., You D., Honnegowda S. (2010). The use of a neonatal mouse model to study respiratory syncytial virus infections. Expert Rev. Anti Infect. Ther..

[8] Saravia J., You D., Shrestha B., Jaligama S., Siefker D., Lee G.I., Harding J.N., Jones T.L., Rovnaghi C., Bagga B. (2015). Respiratory Syncytial Virus Disease Is Mediated by Age-Variable IL-33. PLoS Pathog..

[9] Halim T.Y., Krauss R.H., Sun A.C., Takei F. (2012). Lung natural helper cells are a critical source of Th2 cell-type cytokines in protease allergen-induced airway inflammation. Immunity.

[10] Saenz S.A., Taylor B.C., Artis D. (2008). Welcome to the neighborhood: Epithelial cell-derived cytokines license innate and adaptive immune responses at mucosal sites. Immunol. Rev..

[11] Ohne Y., Silver J.S., Thompson-Snipes L., Collet M.A., Blanck J.P., Cantarel B.L., Copenhaver A.M., Humbles A.A., Liu Y.J. (2016). IL-1 is a critical regulator of group 2 innate lymphoid cell function and plasticity. Nat. Immunol..

[12] Fonseca W., Malinczak C.A., Schuler C.F., Best S.K.K., Rasky A.J., Morris S.B., Cui T.X., Popova A.P., Lukacs N.W. (2020). Uric acid pathway activation during respiratory virus infection promotes Th2 immune response via innate cytokine production and ILC2 accumulation. Mucosal Immunol..

[13] Schuler C.F.t., Malinczak C.A., Best S.K.K., Morris S.B., Rasky A.J., Ptaschinski C., Lukacs N.W., Fonseca W. (2020). Inhibition of uric acid or IL-1beta ameliorates respiratory syncytial virus immunopathology and development of asthma. Allergy.

[14] Yang Y., Wang H., Kouadir M., Song H., Shi F. (2019). Recent advances in the mechanisms of NLRP3 inflammasome activation and its inhibitors. Cell Death Dis..

[15] He Y., Hara H., Nunez G. (2016). Mechanism and Regulation of NLRP3 Inflammasome Activation. Trends Biochem. Sci..

[16] Zahid A., Li B., Kombe A.J.K., Jin T., Tao J. (2019). Pharmacological Inhibitors of the NLRP3 Inflammasome. Front. Immunol..

[17] Bauernfeind F.G., Horvath G., Stutz A., Alnemri E.S., MacDonald K., Speert D., Fernandes-Alnemri T., Wu J., Monks B.G., Fitzgerald K.A. (2009). Cutting edge: NF-kappaB activating pattern recognition and cytokine receptors license NLRP3 inflammasome activation by regulating NLRP3 expression. J. Immunol..

[18] Segovia J., Sabbah A., Mgbemena V., Tsai S.Y., Chang T.H., Berton M.T., Morris I.R., Allen I.C., Ting J.P., Bose S. (2012). TLR2/MyD88/NF-kappaB pathway, reactive oxygen species, potassium efflux activates NLRP3/ASC inflammasome during respiratory syncytial virus infection. PLoS ONE.

[19] Triantafilou K., Kar S., Vakakis E., Kotecha S., Triantafilou M. (2013). Human respiratory syncytial virus viroporin SH: A viral recognition pathway used by the host to signal inflammasome activation. Thorax.

[20] Shim Y.R., Lee H.K. (2015). Caspase-1 independent viral clearance and adaptive immunity against mucosal respiratory syncytial virus infection. Immune Netw..

[21] Coll R.C., Robertson A.A., Chae J.J., Higgins S.C., Munoz-Planillo R., Inserra M.C., Vetter I., Dungan L.S., Monks B.G., Stutz A. (2015). A small-molecule inhibitor of the NLRP3 inflammasome for the treatment of inflammatory diseases. Nat. Med..

[22] Kanneganti T.D., Ozoren N., Body-Malapel M., Amer A., Park J.H., Franchi L., Whitfield J., Barchet W., Colonna M., Vandenabeele P. (2006). Bacterial RNA and small antiviral compounds activate caspase-1 through cryopyrin/Nalp3. Nature.

[23] Lukacs N.W., Moore M.L., Rudd B.D., Berlin A.A., Collins R.D., Olson S.J., Ho S.B., Peebles R.S. (2006). Differential immune responses and pulmonary pathophysiology are induced by two different strains of respiratory syncytial virus. Am. J. Pathol..

[24] Malinczak C.A., Fonseca W., Rasky A.J., Ptaschinski C., Morris S., Ziegler S.F., Lukacs N.W. (2019). Sex-associated TSLP-induced immune alterations following early-life RSV infection leads to enhanced allergic disease. Mucosal Immunol..

[25] Garcia C.G., Bhore R., Soriano-Fallas A., Trost M., Chason R., Ramilo O., Mejias A. (2010). Risk factors in children hospitalized with RSV bronchiolitis versus non-RSV bronchiolitis. Pediatrics.

[26] Reed M., Morris S.H., Owczarczyk A.B., Lukacs N.W. (2015). Deficiency of autophagy protein Map1-LC3b mediates IL-17-dependent lung pathology during respiratory viral infection via ER stress-associated IL-1. Mucosal Immunol..

[27] Terajima M., Yamaya M., Sekizawa K., Okinaga S., Suzuki T., Yamada N., Nakayama K., Ohrui T., Oshima T., Numazaki Y. (1997). Rhinovirus infection of primary cultures of human tracheal epithelium: Role of ICAM-1 and IL-1beta. Am. J. Physiol..

[28] Shi L., Manthei D.M., Guadarrama A.G., Lenertz L.Y., Denlinger L.C. (2012). Rhinovirus-induced IL-1beta release from bronchial epithelial cells is independent of functional P2X7. Am. J. Respir. Cell Mol. Biol..

[29] Piper S.C., Ferguson J., Kay L., Parker L.C., Sabroe I., Sleeman M.A., Briend E., Finch D.K. (2013). The role of interleukin-1 and interleukin-18 in pro-inflammatory and anti-viral responses to rhinovirus in primary bronchial epithelial cells. PLoS ONE.

[30] Cauchois R., Koubi M., Delarbre D., Manet C., Carvelli J., Blasco V.B., Jean R., Fouche L., Bornet C., Pauly V. (2020). Early IL-1 receptor blockade in severe inflammatory respiratory failure complicating COVID-19. Proc. Natl. Acad. Sci. USA.

[31] Van de Veerdonk F.L., Netea M.G. (2020). Blocking IL-1 to prevent respiratory failure in COVID-19. Crit. Care.

[32] Bozzi G., Mangioni D., Minoia F., Aliberti S., Grasselli G., Barbetta L., Castelli V., Palomba E., Alagna L., Lombardi A. (2020). Anakinra combined with methylprednisolone in patients with severe COVID-19 pneumonia and hyperinflammation: An observational cohort study. J. Allergy Clin. Immunol..

[33] Camelo A., Rosignoli G., Ohne Y., Stewart R.A., Overed-Sayer C., Sleeman M.A., May R.D. (2017). IL-33, IL-25, and TSLP induce a distinct phenotypic and activation profile in human type 2 innate lymphoid cells. Blood Adv..

[34] Dinarello C.A. (2018). Overview of the IL-1 family in innate inflammation and acquired immunity. Immunol. Rev..

[35] Barlow J.L., Bellosi A., Hardman C.S., Drynan L.F., Wong S.H., Cruickshank J.P., McKenzie A.N. (2012). Innate IL-13-producing nuocytes arise during allergic lung inflammation and contribute to airways hyperreactivity. J. Allergy Clin. Immunol..

[36] Zhu Z., Homer R.J., Wang Z., Chen Q., Geba G.P., Wang J., Zhang Y., Elias J.A. (1999). Pulmonary expression of interleukin-13 causes inflammation, mucus hypersecretion, subepithelial fibrosis, physiologic abnormalities, and eotaxin production. J. Clin. Investig..

[37] Mahmutovic Persson I., Menzel M., Ramu S., Cerps S., Akbarshahi H., Uller L. (2018). IL-1beta mediates lung neutrophilia and IL-33 expression in a mouse model of viral-induced asthma exacerbation. Respir. Res..

[38] Gschwandtner M., Derler R., Midwood K.S. (2019). More Than Just Attractive: How CCL2 Influences Myeloid Cell Behavior Beyond Chemotaxis. Front. Immunol..

[39] Ajuebor M.N., Flower R.J., Hannon R., Christie M., Bowers K., Verity A., Perretti M. (1998). Endogenous monocyte chemoattractant protein-1 recruits monocytes in the zymosan peritonitis model. J. Leukoc. Biol..

[40] Deshmane S.L., Kremlev S., Amini S., Sawaya B.E. (2009). Monocyte chemoattractant protein-1 (MCP-1): An overview. J. Interferon Cytokine Res..

[41] Stankovic A., Slavic V., Stamenkovic B., Kamenov B., Bojanovic M., Mitrovic D.R. (2009). Serum and synovial fluid concentrations of CCL2 (MCP-1) chemokine in patients suffering rheumatoid arthritis and osteoarthritis reflect disease activity. Bratisl. Lek. Listy.

[42] Harrington J.R. (2000). The role of MCP-1 in atherosclerosis. Stem Cells.

[43] Panee J. (2012). Monocyte Chemoattractant Protein 1 (MCP-1) in obesity and diabetes. Cytokine.

[44] Chen Y., Wang J., Liu C., Su L., Zhang D., Fan J., Yang Y., Xiao M., Xie J., Xu Y. (2020). IP-10 and MCP-1 as biomarkers associated with disease severity of COVID-19. Mol. Med..

[45] Liao J., Kapadia V.S., Brown L.S., Cheong N., Longoria C., Mija D., Ramgopal M., Mirpuri J., McCurnin D.C., Savani R.C. (2015). The NLRP3 inflammasome is critically involved in the development of bronchopulmonary dysplasia. Nat. Commun..

[46] Stouch A.N., McCoy A.M., Greer R.M., Lakhdari O., Yull F.E., Blackwell T.S., Hoffman H.M., Prince L.S. (2016). IL-1beta and Inflammasome Activity Link Inflammation to Abnormal Fetal Airway Development. J. Immunol..

[47] Cornelius D.C., Travis O.K., Tramel R.W., Borges-Rodriguez M., Baik C.H., Greer M., Giachelli C.A., Tardo G.A., Williams J.M. (2020). NLRP3 inflammasome inhibition attenuates sepsis-induced platelet activation and prevents multi-organ injury in cecal-ligation puncture. PLoS ONE.

